# The impact of brace treatment for developmental dysplasia of the hip on caregivers and families: a thematic analysis of literature review and stakeholder survey

**DOI:** 10.1302/2633-1462.68.BJO-2025-0063.R1

**Published:** 2025-08-01

**Authors:** Joanna Craven, Olivia O'Malley, Wesley W. E. S. Theunissen, Daniel C. Perry

**Affiliations:** 1 University of Liverpool, Liverpool, UK; 2 Wales Deanery, Cardiff, UK; 3 Imperial College London, London, UK; 4 Mersey Deanery, Liverpool, UK; 5 Department of Orthopedic Surgery & Trauma, Máxima Medical Center, Veldhoven, The Netherlands

**Keywords:** DDH, brace treatments, Developmental dysplasia of the hip (DDH), randomized controlled trials, dysplasia, clinical outcomes, clinicians, strain, Pavlik harness, arthroplasties, hip joint

## Abstract

**Aims:**

To identify the effect on the family and/or caregivers when infants undergo brace treatment for developmental dysplasia of the hip (DDH) by integrating findings from a literature review and stakeholder survey.

**Methods:**

Thematic analysis combining a comprehensive literature review and data from a UK-based online survey with international involvement. Identification of key themes related to the effect of brace treatment for DDH on the family/ caregivers.

**Results:**

The literature review identified eight relevant articles for inclusion. The online survey had 131 participants. During the thematic analysis, ten key themes emerged, highlighting substantial emotional distress among parents, issues with information provision and consistency, and practical challenges related to clothing, feeding, and sleep. Additional concerns included cleanliness, equipment, child development concerns, infant discomfort, bonding, financial impact, and disruption to daily life.

**Conclusion:**

Brace treatment for DDH has a significant impact on various aspects of family life. Identifying the specific areas of family life affected by brace treatment enables recognition of key challenges, informing the development of robust support systems, clear communication strategies, and customized informational resources. While bracing remains the cornerstone of DDH management in infants, with proven effectiveness in achieving positive clinical outcomes, substantial uncertainties persist regarding critical aspects of treatment, including determining the severity of dysplasia that warrants brace treatment, the optimal duration of treatment, and the most effective approach to brace removal. Resolving these uncertainties requires well-designed randomized controlled trials to establish clear, evidence-based guidelines. Furthermore, evaluations of brace treatments should explicitly incorporate family-centred outcomes. Developing a core outcome set focused on family-relevant measures would significantly enhance the design, comparability, and quality of future DDH research.

Cite this article: *Bone Jt Open* 2025;6(8):851–858.

## Introduction

Developmental dysplasia of the hip (DDH) is characterized by the abnormal development of the hip joint. It is a spectrum of abnormality ranging from an immature hip through to a fully dislocated joint.^1,2^ Approximately 2% to 3% of infants are affected by some degree of hip dysplasia, with 1 in 1,000 presenting with a completely dislocated hip.^3^ Early diagnosis and intervention are essential to prevent long-term complications such as early-onset arthritis.^4,5^ Brace treatment, commonly involving devices such as the Pavlik harness, is the standard non-surgical intervention for DDH in infants.

Although brace treatment is effective for most infants with DDH, there are significant uncertainties and clinical equipoise regarding key aspects of care such determining the severity of dysplasia that warrants brace treatment, the optimal duration of treatment and the most effective approach to brace removal.^6^ This underscores the need for randomized controlled trials to establish evidence-based best practices and achieve standardized care.^7,8^ Very few existing clinical trials have considered the broader impact of brace treatment on the family unit.^9,10^

Brace treatment usually begins when infants are only a few weeks old,^6^ which is a period of significant transition for families adjusting to life with a newborn. Families navigating brace treatment for DDH encounter a range of stressors, from emotional distress upon diagnosis to practical difficulties managing brace-related tasks.^9,11-16^ Understanding the impact on the family unit is essential for developing comprehensive care strategies that address the holistic needs of affected families.

While individual qualitative studies have explored the experiences of caregivers during the period of brace treatment,^11-17^ there has yet to be a comprehensive synthesis of these findings. Such a synthesis is essential to provide a deeper, more holistic understanding of the challenges, perceptions, and support needs of caregivers throughout this treatment process.

This study aims to conduct a thematic analysis that integrates findings from a literature review and a UK-based online survey to highlight the effect that this treatment period has on these families/caregivers.

## Methods

A mixed-methods approach was adopted, integrating existing qualitative literature supplemented by primary survey data.

### Literature review

A systematic literature review was carried out to identify family-centred outcomes during brace treatment for DDH. The search strategy was predefined and used a systematic approach to extract relevant studies. The search strategy is outlined in Table I.

**Table I.** Population, exposure, and outcome (PEO) search strategy for literature review.

Population	“developmental dysplasia of the hip” OR “DDH” OR “CDH” OR “congenital dysplasia of the hip” OR “hip dysplasia”
Exposure	“brace” OR “splint” OR “harness” OR “Pavlik harness” OR “removable rigid splint”
Outcome	“family centred outcomes” OR “family focused outcomes” OR “family experiences” OR “patient experience” OR “parent experience” or "experiences of parents"

To develop the search, two key articles were identified: ‘Exploring the experiences of parents caring for their infant with developmental dysplasia of the hip (DDH): an interpretative phenomenological analysis’,^11^ and ‘Parental experiences of children with developmental dysplasia of the hip: a qualitative study’.^12^ The search terms were designed to be sufficiently sensitive to identify the two known relevant articles, as well as any additional publications.

The literature review included any articles of published or unpublished qualitative work and theses, examining the family experience during DDH treatment. Inclusion criteria were articles specific to DDH and the family perception/ experience. Articles were only included if available in English. Exclusion criteria included any articles that explored family experience of DDH treatment where the treatment was exclusively surgical.

The search strategy used was identified for three electronic databases: MEDLINE (1946 to 30 April 2024); CINAHL (Cumulative Index to Nursing and Allied Health Literature; 1937 to 30 April 2024), and ProQuest (all available years, searched 30 April 2024). Electronic databases were limited by English language and human studies. Databases were searched on 30 April 2024. Reference lists of included articles were checked to identify any additional studies. This approach accommodated the broad scope and varied formats of the previously identified articles, which included a doctoral thesis and a scientific paper.

Two reviewers (both clinician researchers) independently screened articles (OO, JC). The relevant articles were read, and data were extracted regarding the year of publication, study design, study location, study size, and their sample characteristics. Impacts on the family were tabulated as ‘family-centred outcomes’ with relevant supportive information. The authors collaboratively agreed on the groupings of the outcomes into themes.

### UK-based online survey with international involvement

An online survey was developed to capture the perspectives of parents and clinicians/researchers on family-centred outcomes for infants undergoing brace treatment for DDH. The survey was specifically designed to capture additional outcomes potentially missed by previous qualitative studies. The survey included an open-ended question designed to elicit comprehensive data on various aspects of the DDH treatment experience, including any that were not identified through the literature search.

The survey was designed in conduction with a parent representative and supported by the STEPs worldwide charity.^18^ The survey was shared on social media (X, Instagram, and Facebook) through collaboration with the STEPs charity and circulated to professional networks.

Participants were eligible to take part if they were the caregiver (parent or legal guardian) of a child who was treated for DDH in a brace, a researcher involved in DDH research, or a clinician involved in DDH care.

The survey was conducted using Googleforms (Google, USA) over one month in summer 2024. All participants were provided with a participant information sheet and consented to participate via an online form. Participants were asked to provide their role and country of residence.

Caregivers were asked “Please think about when your child was wearing a brace (such as a Pavlik harness) for DDH, how did it affect you as parents, the family unit and the infant? Please list as many outcomes as you can think of which affect the family when a infant is treated for DDH in a brace.”

Clinicians and researcher were asked “Please think about when you are involved in the care/research of a infant treated in a brace for DDH. What do you see as important for the family during this time? Please list as many outcomes as you can think of.”

### Ethical considerations

The study received ethical approval from University of Liverpool as part of a broader study to develop a family-centred core outcome set for infants undergoing brace treatment for DDH. Informed consent was obtained from all participants and data were anonymized to ensure confidentiality.

## Results

### Literature search

**Fig. 1:**
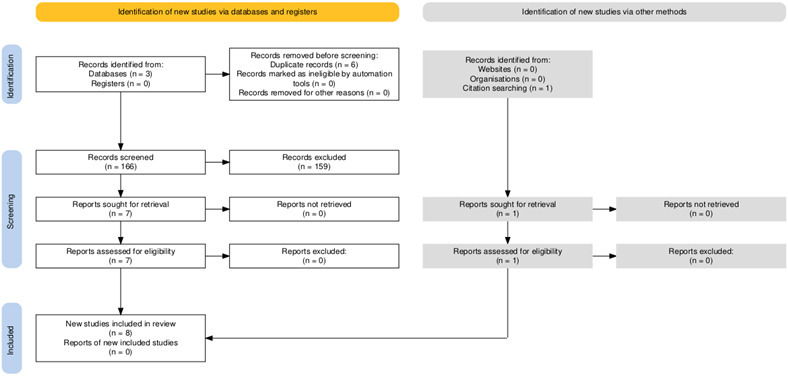
PRISMA flow chart.

The initial search of databases identified 172 articles. Six duplicate articles were removed. Figure 1 displays the PRISMA flow diagram.^19^ Two reviewers (JC, OO) independently screened the title and abstract of 166 articles against inclusion and exclusion criteria. Three disagreements were reviewed by the reviewers and consensus reached. Seven articles were identified for full text review. The references of the seven included articles were screened and one additional relevant article was identified and included from searching the reference lists of the included papers. The characteristics of the included studies are listed in Table II.

**Table II.** Summary of qualitative studies identified in literature search.

Study	Year of publication	Design	Location	Study size	Sample
Hassan et al^17^	2009	Cohort survey and structured interviews	Jordan	160	Parents of children treated for DDH in a Pavlik harness
Poole^11^	2019	Semi-structured interviews	UK	18	Parents of infants with DDH
Wakely et al^16^	2020	Semi-structured interviews	Australia	7	Parents of children with DDH
Gibbard et al^13^	2021	Cross-sectional survey	International online survey	739	Parents/guardians of children treated for DDH DDH patients aged > 18 years
Theunissen et al^12^	2022	Semi-structured interviews	Netherlands	22	Parents of children aged less than one year treated for DDH in a Pavlik harness
Grzybowski et al^14^	2023	Cross-sectional survey	International online survey	530	Parents/ guardians of children treated for DDH in an orthotic
Hoffer et al^9^	2023	Retrospective comparative cross-sectional survey	International online survey	50 (plus 30 participants from a control group)	Mothers of infants treated for DDH in a Pavlik harness; plus control group of mothers of infants who were screened for DDH only
Harry et al^15^	2022	Cross-sectional survey	Australia	753	Parents/guardians of children with DDH

DDH, developmental dysplasia of the hip.

Initially, 12 outcomes were identified through the literature search, which are parental wellbeing, information and resources, clothing, equipment, feeding, sleep, cleanliness, child development, skin irritation, infant discomfort, bonding, and financial impact. A summary of the outcomes extracted from the included studies, along with associated quotes and additional information, is provided in the Supplementary Material.

### Online survey

The survey received responses from 131 participants, predominantly mothers residing in the UK. Participant role (Table III) and country where they live (Table IV) was collected.

**Table III.** Participants by role.

Role	Participants, n (%)
Mother	116 (88.5)
Father	2 (1.5)
Paediatric orthopaedic surgeon	7 (5.3)
Nurse	4 (3.1)
Physiotherapist	1 (0.8)
Researcher	1 (0.8)
Total	131 (100)

**Table IV.** Participants by country.

Country	Participants, n (%)
UK	102 (78.0)
USA	11 (8.4)
Australia	5 (3.8)
The Netherlands	4 (3.1)
Ireland	3 (2.3)
Channel Islands	3 (2.3)
New Zealand	1 (0.8)
South Africa	1 (0.8)
Brazil	1 (0.8)
Total	131 (100)

The 12 outcomes identified in the literature search were also identified through the online survey. One additional outcome ‘disruption to daily life’ was also identified. These 13 outcomes are outlined in Table V.

**Table V.** Outcomes reported in survey.

Outcomes	Survey respondents who reported, n (%)
**Parental wellbeing**	100 (76)
General wellbeing	34 (26)
Feeling overwhelmed by diagnosis	31 (24)
Reactions from others	25 (19)
Concerns about the future	16 (12)
Managing with infant in brace	12 (9)
Parental isolation	12 (9)
Marital stress	4 (3)
**Information and resources**	51 (39)
Lack of support	24 (18)
Lack of Information	18 (14)
Inconsistent information	7 (5)
Poorly given information	4 (3)
Clothing	66 (50)
**Equipment**	58 (44)
Car seats	25 (19)
Prams	10 (8)
Cots/Moses baskets	7 (5)
Carriers/wraps	7 (5)
Other equipment	1 (< 1)
Feeding	62 (47)
Sleep	24 (18)
**Cleanliness**	110 (84)
Nappy changes	35 (27)
Bath time	35 (27)
General issues	16 (12)
**Child development concerns**	16 (12)
Tummy time	12 (9)
Walking	4 (3)
Attachment issues	3 (2)
Introducing food (weaning)	3 (2)
Skin irritation	16 (12)
**Infant discomfort**	37 (28)
General discomfort	28 (21)
Digestive issues	9 (7)
**Bonding**	30 (23)
General bonding concerns	16 (12)
Cuddling	14 (11)
**Financial impact**	18 (14)
Cost of equipment	9 (7)
Unable to go to work	4 (3)
General financial concerns	4 (3)
**Disruption to daily life**	30 (23)
Burden of hospital attendances	16 (12)
Day to-day life	14 (11)
Swimming and activities	4 (3)

### The thematic analysis

The thematic analysis identified ten key themes derived from 13 outcomes revealed through the literature review and online survey. These themes include parental wellbeing, access to information and resources, practical challenges, equipment, cleanliness, child development, infant discomfort, bonding, financial impact, and disruptions to daily life. Representative quotes were used to illustrate key themes (Table VI).

**Table VI.** Key themes and representative quotes on family impact.

Theme	Quotes
Parental wellbeing	“I found the experience challenging, upsetting and stressful” “It was really traumatic for me as a mother as I felt helpless” “It was a very scary diagnosis for any parent, but as a first time parent I felt very overwhelmed initially, and it took a while to find our groove. I knew it wasn't life or death, but I didn’t know how successful the harness would be, how long it would take before we had the reassurance we needed, and whether or not she required surgery in the future” “Honestly one of the hardest times of my life”
Information provision	“Found lots of mixed messages on what can and can’t be done once the brace is removed, I took the advice from my consultant, but it was clear different consultants had different opinions” “We were not fully informed about how to hold, feed and transport our infant while in harness. We were not provided with any information regarding equipment, clothing, washing. Midwives did not seem to have knowledge of DDH, so were unhelpful when asked questions especially about breast feeding positions” “All the info I had was from online research rather than from the hospital. It would have been good to have been better prepared as to what to expect etc.”
Practical challenges (clothing, feeding, and sleep)	“Breast feeding the infant was stressful and uncomfortable for us both” “A lot of the clothes we'd bought she couldn't wear, bought before birth after birth and as presents” “We struggled to find suitable clothing, I know in hindsight this is a very superficial issue but her whole three to six and six to nine month wardrobe was wasted because we had to purchase specialist DDH clothing and at the time this range was extremely limited” “Unable to wear all the lovely clothes I had been given as gifts” “Concern around sleep was the biggest worry” “She was so irritable - wouldn’t sleep for hours”
Cleanliness	“On top of learning how to deal with a newborn for the first time, we also had to navigate changing a infant with a harness on and dealing with nappy explosions. We were unable to bath her for a long time as she was in the harness 24/7 so I feel we missed out on that lovely experience” “Little help changing because didn’t know how with harness” “Hospital trip if her nappy leaked to have a clean harness fitted” “Bath time was a nightmare in those first few weeks as we had to rush” “Harness cannot fully be cleaned as it is handwashing only and she has to wear it 23 hours a day”
Equipment	“Difficulty finding safe equipment to use, such as car seats, highchairs, prams” “Finding a suitable car seat. Not fitting in supermarket trolleys. I had to carry her in a carrier till she was 9 months old when I went shopping.” “Required the expense of a new car seat specifically for DDH”
Child development concerns	“Unable to perform tummy time well” “We are convinced it contributed to some issues he had around attachment” “Infant was somewhat delayed in large motor milestones like crawling and walking” “Delayed milestones”
Infant discomfort	“I struggled to sleep as I felt like she would be uncomfortable and watched her a lot” “Not being able to rub her tummy in the usual way you would for a infant to alleviate the normal tummy pains of a infant meant tummy cramps were more of an issue”
Bonding	“it [brace treatment for DDH] lead to me falling into a depression and feeling very detached from her” “Bonding - huge impact on my relationship building with her” “it was horrible for me to feel like there was a barrier between me and my infant when we are supposed to be skin to skin”
Financial impact	“A lot of extra expense” “Expenses, like buying a infant wear wrap and beanbag, so that she would be comfy” “regular scans and consultant appointments meant requiring childcare for other child and expenses (fuel, parking)”
Disruption to daily life	“I was sad to miss out on activities, like infant massage and swimming, that I had enjoyed with my older son” “The limitations to get and about to see family and friends”

- **Parental wellbeing:**
  Parental wellbeing was significantly affected across multiple dimensions, impacting mental health and daily functioning. Many parents reported feelings of being overwhelmed and unprepared following the initial diagnosis, accompanied by distress related to intrusive questioning from others, and concerns about their child’s long-term prognosis. Daily management of an infant in a brace further intensified feelings of social isolation and placed considerable strain on marital relationships.^9,11-13,15-17^
- **Information and resources:**
  Access to adequate information and support emerged as a critical challenge. Parents frequently described receiving insufficient or conflicting advice from healthcare providers and experiencing ineffective or unclear communication regarding DDH management. Such inconsistencies in information provision exacerbated stress levels and contributed to mistrust.^9,11-13,15-17^ Further qualitative research has been undertaken to explore information provision for DDH parents.^20^
- **Practical challenges:**
  Significant practical difficulties were categorized into three sub-themes: clothing, feeding, and sleep. Finding appropriate clothing compatible with the brace was problematic, causing frustration and financial strain.^11-17^ Feeding routines, particularly breastfeeding, were disrupted by brace management, increasing stress and leading to earlier cessation.^11-14,16^ Supporting evidence includes a Swedish study that reported significantly lower breastfeeding rates among infants prescribed a von Rosen splint compared with healthy controls.^21^ Sleep disturbances related to brace use adversely affected both infant and parental wellbeing.^16^
- **Equipment:**
  Challenges associated with equipment included issues related to car seats, prams, sleeping arrangements (cots or Moses baskets), and carriers or wraps. Parents struggled to find brace-compatible equipment, leading to practical inconveniences and safety concerns. Limited suitable options often required additional purchases or adaptations, further complicating caregiving tasks.^12-14,16^
- **Cleanliness:**
  Parents managing DDH treatment experienced significant cleanliness-related challenges, which were categorized into three sub-themes: nappy changes, bath time, and general hygiene. Nappy changes became particularly complicated due to the brace, with some parents requiring additional hospital visits for brace adjustments or arthroplasties following these difficulties. Bathing infants wearing braces posed further practical challenges; in cases where bathing was not feasible, parents encountered both practical inconveniences and emotional distress. Additionally, parents consistently expressed ongoing concerns regarding maintaining the cleanliness and overall hygiene of the brace itself, emphasizing that hygiene management significantly disrupted daily routines for both parents and infants.^9,11,14,15,17^
- **Child development concerns:**
  Parents expressed several concerns regarding developmental milestones, categorized into sub-themes: tummy time, walking, attachment issues, and introducing food (weaning). Parents commonly reported difficulty with tummy time and concern that this would adversely affect their child. They worried about the impact of brace use on achieving physical milestones and future walking ability. Although these worries were prominent among parents, literature indicates limited evidence of long-term developmental issues associated with brace use.^9,11,12,15^
- **Infant discomfort:**
  Concerns related to infant comfort included general discomfort, skin irritation and digestive issues. Worries focused on the infant’s physical comfort while wearing the brace, with particular attention to skin irritation, reflux and digestive discomfort. Literature suggests that although infants often tolerate a brace well, parental anxiety frequently remains high.^9,11,12,14,16,17^ Additionally, prolonged brace use has been associated with skin irritation.^11,14,17^
- **Bonding:**
  Challenges related to bonding included general concerns about emotional connection and physical difficulties cuddling or holding the child due to the brace. Parents worried that the harness could negatively affect their relationship and closeness with the infant. Parents highlighted that the physical barrier of the brace can impede traditional bonding activities and affect bonding.^9,11-16^
- **Financial impact:**
  Financial strain arose from the cost of specialized equipment, loss of income due to caregiving responsibilities and additional expenses associated with managing DDH, significantly contributing to overall stress.^12^
- **Disruption to daily life:**
  Daily life was disrupted by frequent hospital visits, substantial adjustments to family routines, and restrictions on recreational and social activities, such as swimming or group events, leading to logistical challenges and changes in family dynamics.

## Discussion

This thematic analysis summarizes the extensive impact of DDH brace treatment on families. Although qualitative studies on this topic exist, no single study has comprehensively addressed all outcomes identified within this article.

These findings are consistent with existing literature highlighting the emotional toll of chronic paediatric conditions on parents.^12,13,22^ Information and resources were identified as critical areas needing improvement. The lack of consistent, high-quality information leads to confusion and increased parental anxiety. This aligns with previous studies emphasizing the need for better communication and support systems in chronic disease management.^11,13,15^ Parents often obtain unfiltered and inconsistent internet advice of varied quality and accuracy.^23^ Qualitative work has been done to explore information provision for parents whose infants undergo brace treatment for DDH.^20^ Inconsistent clinical practices contribute to the mixed information parents receive both from healthcare providers and online sources. Standardizing care pathways is essential to ensure that parents receive clear, consistent information.

Our study has limitations. The survey was designed and reviewed by a parent representative and the STEPs charity, but did not undergo pilot testing. The online survey relied on a single open-ended question for outcome reporting, rather than prompting participants with a predefined list. While this approach allowed parents to freely express the full range of outcomes affecting their families, it may have resulted in lower reporting rates compared with structured surveys that confirm experiences for each outcome. The decision not to list specific outcomes was made to avoid leading responses and potential bias.

While the literature review included international studies, survey responses primarily reflected a UK perspective, which may limit the findings’ generalizability to healthcare systems with different practices, resources, and cultural norms. Additionally, both the survey and literature search had low representation of fathers, which may result in an incomplete understanding of family dynamics and parental experiences, potentially skewing findings toward maternal perspectives. Families who choose to participate in surveys are often those with strong opinions, introducing the possibility of voluntary response bias. As with all self-reported survey data, there is a potential for response biases, such as social desirability or recall bias, which could affect the accuracy and reliability of the reported outcomes. Clinicians were invited to participate, recognizing their potential to identify outcomes observed through their interactions with families. However, clinician engagement in this study was minimal.

The findings highlight several areas for intervention:

- **Emotional support services:** Implementing support programmes, such as counselling and support groups, to address the emotional burden on parents.
- **Standardized care protocols:** Establishing evidence-based care protocols to reduce inconsistencies in treatment advice and management strategies. Ensuring family-centred outcomes are considered in future randomized controlled trials.
- **Enhanced information provision:** Developing standardized, high-quality informational resources to ensure consistency and reliability of information provided to families. Information should be written at a reading level that is understandable for all parents.
- **Practical assistance:** Providing guidance and resources for managing practical challenges, including clothing and equipment adaptations.
- **Financial support:** Exploring avenues for financial assistance or subsidies to mitigate the economic impact on families.

Future studies should focus on developing and validating a core outcome set for family-centred outcomes during brace treatment for DDH, to ensure that the effects of the treatment on families can be measured in a reliable, consistent way. Longitudinal research could explore the long-term impact of DDH treatment on family dynamics and child development. Studies aimed at improving information provision and emotional support are also warranted. Randomized controlled trials to determine evidence-based standardized care protocols for brace wearing in DDH are needed.^6^

This thematic analysis reveals that managing DDH brace treatment significantly affects multiple aspects of family life, particularly parental wellbeing, access to information, and practical daily challenges. Addressing these areas through targeted interventions and improved support systems is essential. Integrating these findings into clinical practice can lead to more holistic and effective care for families navigating brace wearing for DDH.

**Take home message**

- This study integrates qualitative data from an online survey with existing literature, providing an overview of the effect of brace treatment for developmental dysplasia of the hip on the families.

## Data Availability

The data that support the findings for this study are available to other researchers from the corresponding author upon reasonable request.

## References

[1] Dunn PM The anatomy and pathology of congenital dislocation of the hip Clin Orthop Relat Res 1976 119 23 27 954316

[2] Dezateux C Rosendahl K Developmental dysplasia of the hip Lancet 2007 369 9572 1541 1552 10.1016/S0140-6736(07)60710-7 17482986

[3] Sewell MD Rosendahl K Eastwood DM Developmental dysplasia of the hip BMJ 2009 339 b4454 10.1136/bmj.b4454 19934187

[4] Singh A Wade RG Metcalfe D Perry DC Does this infant have a dislocated hip?: the rational clinical examination systematic review JAMA 2024 331 18 1576 1585 10.1001/jama.2024.2404 38619828

[5] Vaquero-Picado A González-Morán G Garay EG Moraleda L Developmental dysplasia of the hip: update of management EFORT Open Rev 2019 4 9 548 556 10.1302/2058-5241.4.180019 31598333 PMC6771078

[6] Dwan K Kirkham J Paton RW Morley E Newton AW Perry DC Splinting for the non-operative management of developmental dysplasia of the hip (DDH) in children under six months of age Cochrane Database Syst Rev 2022 10 10 CD012717 10.1002/14651858.CD012717.pub2 36214650 PMC9549867

[7] Westacott DJ Perry DC The treatment of neonatal hip dysplasia with splints in the United Kingdom: time for consensus? J Child Orthop 2020 14 2 112 117 10.1302/1863-2548.14.190156 32351623 PMC7184644

[8] No authors listed Paediatric Trauma and Orthopaedic Surgery Getting It Right First Time (GIRFT) 2024 https://gettingitrightfirsttime.co.uk/surgical_specialties/paediatrictrauma-and-orthopaedic-surgery/ date last accessed 15 July 2025

[9] Hoffer A Chhina H Mulpuri K Cooper AP Early investigation and bracing in DDH impacts maternal wellbeing and breastfeeding J Pediatr Orthop 2023 43 1 e30 e35 10.1097/BPO.0000000000002274 36190923

[10] Gardner F Dezateux C Elbourne D et al. The hip trial: psychosocial consequences for mothers of using ultrasound to manage infants with developmental hip dysplasia Arch Dis Child Fetal Neonatal Ed 2005 90 1 F17 24 10.1136/adc.2002.025684 15613565 PMC1721817

[11] Poole C Edinburgh Napier University Exploring the experiences of parents caring for their infant with Developmental Dysplasia of the Hip (DDH): an interpretative phenomenological analysis

[12] Theunissen W. W. E. S. van der Steen MC van Veen MR van Douveren F Witlox MA Tolk JJ Parental experiences of children with developmental dysplasia of the hip: a qualitative study BMJ Open 2022 12 9 e062585 10.1136/bmjopen-2022-062585 36153020 PMC9511546

[13] Gibbard M Zivkovic I Jivraj B et al. A global survey of patient and caregiver experiences throughout care for developmental dysplasia of the hip J Pediatr Orthop 2021 41 6 e392 e397 10.1097/BPO.0000000000001813 34096547 PMC8183474

[14] Grzybowski G Bliven E Wu L et al. Caregiver experiences using orthotic treatment options for developmental dysplasia of the hip in children J Pediatr Orthop 2023 43 2 105 110 10.1097/BPO.0000000000002312 36607922 PMC9812410

[15] Harry A Johnston C Twomey S Wakely L A survey of parents’ and carers’ perceptions of parenting a child with developmental dysplasia of the hip Pediatr Phys Ther 2022 34 3 328 333 10.1097/PEP.0000000000000917 35639555

[16] Wakely L Easey P Leys J Johnston C Exploring the lived experience of parenting a child with developmental dysplasia of the hip Phys Occup Ther Pediatr 2021 41 5 503 514 10.1080/01942638.2020.1867694 33557686

[17] Hassan FA Compliance of parents with regard to Pavlik harness treatment in developmental dysplasia of the hip J Pediatr Orthop B 2009 18 3 111 115 10.1097/BPB.0b013e32832942f7 19318986

[18] No authors listed www.stepsworldwide.org date last accessed 23 July 2025

[19] Haddaway NR Page MJ Pritchard CC McGuinness LA *PRISMA2020*: an R package and Shiny app for producing PRISMA 2020-compliant flow diagrams, with interactivity for optimised digital transparency and open synthesis Campbell Syst Rev 2022 18 2 e1230 10.1002/cl2.1230 36911350 PMC8958186

[20] Theunissen W. W. E. S Van der Steen MC Van Veen MR Van Douveren FQMP Witlox MA Tolk JJ Strategies to optimize the information provision for parents of children with developmental dysplasia of the hip Bone Jt Open 2023 4 7 496 506 10.1302/2633-1462.47.BJO-2023-0072.R1 37402475 PMC10319458

[21] Elander G Breast feeding of infants diagnosed as having congenital hip joint dislocation and treated in the von Rosen splint Midwifery 1986 2 3 147 151 10.1016/s0266-6138(86)80006-7 3640996

[22] Cousino MK Hazen RA Parenting stress among caregivers of children with chronic illness: a systematic review J Pediatr Psychol 2013 38 8 809 828 10.1093/jpepsy/jst049 23843630

[23] Fabricant PD Dy CJ Patel RM Blanco JS Doyle SM Internet search term affects the quality and accuracy of online information about developmental hip dysplasia J Pediatr Orthop 2013 33 4 361 365 10.1097/BPO.0b013e31827d0dd2 23653022

